# Iowa State Veterinary Medical Association

**Published:** 1890-12

**Authors:** S. Stewart

**Affiliations:** Secretary


### SOCIETY PROCEEDINGS.

*Iowa State Veterinary Medical Association.—First Day*—The regular annual meeting of the Iowa State Veterinary Medical Association was called to order in the parlors of the Savery House, De Moines, Iowa, at 10 A. M., November 13th, 1890. Dr. L. A. Thomas was made President *pro tem.*, the Presidents and Vice-presidents being absent. Members present: Drs. Thomas, Morse, Inger, R. C. Sayers, E. E. Sayers, S. H. Johnson, M. E. Johnson, G. A. Johnson, Campbell, Brown, Ovens, Stalker, Howell, Lawson, Buffington, Thurtle and Stewart, guests; Drs. Edwards, Nelson, J. W. Williams, Fuller, Simcoke, Stark, Hall, Booth, Ashworth, Platt, Brown, Norton, Derwent, Reynolds, Gibson, Emery and W. L. Williams, of Bloomington, Ill.

After completion of routine business, the hearing of the case against J. A. Campbell, for violation of professional ethics, was called. Dr. Campbell stated that he was not guilty; his name had been used without his knowledge or consent. A resolution was adopted exonorating Dr. Campbell if he filed certain statements by the parties who used his name.

*Afternoon Session*—The Secretary read several communications from absent members; also a letter from W. Horace Hoskins, Secretary United States Veterinary Medical Association, calling especial attention to ethics, and a letter from Prof. A. Liautard, making propositions to furnish our society with reprints of our proceedings and papers.

Ethics were discussed at some length when a motion by the Secretary, duly seconded, creating a committee to present a specific code of ethics for consideration was carried. Drs. Morse, Stalker and Stewart were appointed such committee. A code in harmony with that of the United States Veterinary Medical Association was adopted.

A motion, duly seconded, to accept the offer of Prof. A. Liautard to supply our society with reprints of our proceedings and papers was carried.

Drs. R. C. Sayers and T. A. Brown were appointed to fill vacancies in the Board of Censors. Drs. S. H. Johnson, H. Ovens and J. D. Inger were appointed Auditing Committee to examine the Treasurer's accounts.

The association adjourned to meet at Dr. Morse's infirmary, where several interesting cases were seen and several clinical demonstrations witnessed by the members. Among the cases was a man who had suffered extensive lacerations of the perineum, but which was now completely healed, a result obtained by surgical interference ; a case of odontal tumors in a four-year-old gelding ; bursattee in a stallion ; rapid anæsthesia by the use of the Carlisle muzzle was demonstrated, and the details of its use and management explained by Dr. Thomas ; Dr. Morse exhibited the good qualities of an operating table recently obtained.

*Evening Session*—The Board of Censors reported favorably upon all applications for membership submitted, A motion was carried instructing the Secretary to cast the ballot of the Association for each of the applicants found worthy of membership. The Secretary cast the ballot as instructed, and the following named gentlemen were duly elected to membership : F. H. P. Edwards, E. T. Hall, A. E. Derwent, John McBirney, J. M. Stark, J. C. Norton, J. W. Williams, C. A. Ashworth, J. I. Gibson, S. B. Nelson, L. E. Booth, J. J. McLaughlin, J. O. Simcoke, John E. Brown, R. R. Hammond, Q. C. Fuller, J. H. Platt, and Jas. Hansen.

Dr. Stewart, chairman of the Committee on Collective Statistics, submitted a report ; the committee continued another year. The report disclosed the lack of general interest upon the part of the membership in this phase of scientific research ; only fifty notes on heredity were forwarded to the committee during the past year. The notes received were carefully analyzed. A spirited discussion was engendered by this report. Prof. M. Stalker presented his paper on cystic calculi, and the operative procedure for their removal. This paper was illustrated by several specimens of calculi. The Association adjourned to the banquet hall of the Savery House, and did full justice to a bountiful spread. Prof. Stalker proved himself an accomplished toast-maker, and the entire programme was highly enjoyed by all.

*Second day.—Morning Session*—The Association came to order at 9 A. M., Dr. Thomas, President *pro tem.*, presiding. The Auditing Committee reported the Treasurer's accounts correct and were discharged. On motion by Dr. G. R. Johnson, a vote of thanks was tendered Prof. A. Liautard for the extra number of the *Veterinary Review.* The report of the Committee on Legislation was submitted by Dr. Thomas, chairman. This report is based upon letters from veterinarians located in all parts of Iowa, and recommends an effort to secure legislation governing the practice of veterinary science, the law to provide for the examination of the non-graduate who desires to practice in Iowa. This report was received, the committee increased to five and continued another year. Pre ident Tait Butler, finding it impossible to be present at this meeting, forwarded his address to be presented by the Sec. retary. In his address the President deplored the lack of interest of Iowa men in the United States Veterinary Medical Association, which convened so near to us this year, and comments quite freely upon the scarcity of scientific papers recorded to Iowa's credit during the year just past. He exhorts the members to take one or both American veterinary journals and an European journal if they can, and suggests that our members contribute at least six papers to each of our journals this year. This address received a thorough discussion, and a voluntary agreement on the part of several members to prepare papers for the journals during the coming year was entered on the records. Dr. L. W. Williams read a very valuable paper on odontones, and illustrated his paper by exhibiting many specimens of tooth tumors. Dr. Brown's paper was postponed one year. Dr. G. A. Johnson read a paper on corn-stalk disease. Dr. S. B. Nelson presented notes on the use of eserine as a cathartic. Dr. Edwards presented a paper on a disease known by some as scrofulous ostitis. All the papers received a generous discussion.

The following is the list of officers selected: President, Dr. L. A. Thomas; first vice-president, Dr. A. B. Morse; second vice-president, Dr, G. A. Johnson; secretary and treasurer, Dr. S. Stewart. On motion by Dr. Morse, seconded by Dr. M. E. Johnson, Dr. L. W. Williams was elected an honorary member of the Association, and tendered a vote of thanks for his valuable paper. A vote of thanks was tendered Drs. Thomas, Morse and Howell for the clinical demonstrations given the Society.

Adjourned to meet in Des Moines during the autumn of 1891, the time to be fixed by the President.

S. STEWART, *Secretary.*

